# Flexural Strength and Clinical Classification of Different Layers in 4/5Y-PSZ Zirconia Materials

**DOI:** 10.3390/jfb17060300

**Published:** 2026-06-16

**Authors:** Ulrich Lohbauer, Margit Schwarz, Renan Belli

**Affiliations:** Research Laboratory for Dental Biomaterials, Department for Operative Dentistry and Periodontology, Friedrich-Alexander-Universität Erlangen-Nürnberg (FAU), Glueckstrasse 11, 91054 Erlangen, Germany

**Keywords:** multilayer zirconia, 4/5Y-PSZ, flexural strength, Weibull statistics, fractography, clinical indication

## Abstract

Multilayer 4Y/5Y-PSZ zirconia materials have been developed to combine strength and translucency in monolithic “all-in-one” dental restorations. This study evaluated the flexural strength of different layers (incisal, transition, and dentin) in four commercially available multilayer zirconia systems using three-point bending tests in accordance with ISO 6872. A total of 360 CAD/CAM-fabricated bar-shaped specimens were prepared from the materials CE (Cercon yo ML, DentsplySirona), KA (Katana YML, Kuraray Noritake), PZ (3D ProZir, Aidite), PE (IPS e.max ZirCAD Prime esthetic), and assigned to layer-specific groups based on their position within the discs. After sintering and standardized surface finishing, specimens were tested under three-point bending conditions. Fracture strength was calculated and statistically analysed. Microstructural and fractographic analyses were performed to assess grain structure and to identify fracture origins. The results demonstrated significant differences in flexural strength both among materials and between layers. In general, dentin layers exhibited the highest strength, reaching mean values up to 1143 MPa, while incisal layers showed significantly lower values, with minima around 572 MPa. Only one material (CE) maintained flexural strength above the ISO threshold of 800 MPa across all layers, qualifying for unrestricted (class 5) clinical use. Other materials showed limitations, particularly in the more translucent incisal regions (KA, PE). One material fell below the ISO threshold (PZ). Weibull moduli revealed differences in reliability, with moduli ranging from 4.7 to 16.5. Fractographic evaluation identified typical fracture patterns such as surface grinding defects and internal porosity, but no abnormal fracture origins. The strength gradient corresponds to microstructural differences, particularly grain size and phase composition, influenced by yttria content. Increased translucency in incisal layers is associated with reduced mechanical performance. These findings emphasize that, despite aesthetic advantages, layer-dependent strength variations must be considered when selecting multilayer zirconia for clinical applications, especially in long-span restorations.

## 1. Introduction

All-ceramic materials have become a cornerstone of contemporary prosthetic dentistry due to their excellent biocompatibility, chemical stability, and favourable esthetic properties [1]. Among these materials, zirconia-based ceramics derived from zirconium dioxide have gained widespread clinical acceptance for the fabrication of fixed dental prostheses. Zirconia’s combination of high fracture resistance (strength and toughness), chemical inertness, and compatibility with digital manufacturing workflows has made it one of the most widely used ceramic materials in restorative dentistry. Over the past two decades, continuous material innovations have transformed zirconia from an opaque framework ceramic into a versatile class of monolithic restorative materials often described as all-in-one zirconia, capable of simultaneously fulfilling mechanical and esthetic requirements [2,3,4].

Despite their favourable mechanical properties, early 3Y-TZP (3 mol% yttria-stabilized tetragonal zirconia polycrystal) zirconia systems were limited by their relatively high opacity. For this reason, zirconia frameworks were traditionally veneered with esthetic veneering ceramics to reproduce the optical appearance of natural teeth. However, veneering fractures soon became one of the most frequently reported technical complications of zirconia-based restorations, particularly in posterior fixed dental prostheses [5,6].

In response to these limitations, the concept of monolithic zirconia restorations became popular among practitioners, and, as a consequent solution, material development focused on combining the superior mechanical performance of strong 3Y-TZP with the esthetic appearance of a more translucent microstructure at higher yttria concentrations (e.g., 5 mol% yttria-partially stabilized zirconia (5Y-PSZ)) [7,8].

Today, zirconia materials frequently employ multilayer or gradient designs, in which layers of different yttria concentrations or pigment distributions are incorporated within a single CAD/CAM blank. This strategy allows for the reproduction of the natural colour gradient of teeth, with higher translucency in incisal regions and greater strength in cervical regions. Such materials are often described as all-in-one zirconia, reflecting their ability to combine strength, translucency, and colour characteristics within a single monolithic restoration [8].

Following the all-in-one concept, multilayered zirconia materials are now widely used for a broad range of prosthetic indications and continue to play an increasingly important role in contemporary restorative dentistry [9].

The clinical success and longevity of long-span prosthetic constructs are guided by the material’s intrinsic properties as well as by the specific bridge design [10]. The ultimate proof of concept is still based on evidence from long-term clinical trials [5,6]. However, as clinical proof is comparably laborious and time-consuming to achieve, pre-clinical testing is a valid approach in current material development [11,12]. Testing protocols focused on high clinical relevance and significance on the one side and a high level of standardization on the other side are key to the field of pre-clinical assessment of prosthetic materials. Among various material-related parameters, fracture strength testing is one of the most standardized procedures, which allows for clinical indications to be predicted and recommended [13]. ISO, as an internationally agreed consensus on procedures, provides the envelope for measuring the fracture strength of dental ceramics. The protocol for flexural strength testing is generally outlined in ISO 14704 [14] and specifically tailored for dentistry in ISO 6872 [15], along with a strength- (and toughness-) based assignment of prosthetic materials to clinical indications. Regarding high-performance zirconia materials, ISO guides its clinical use as a “monolithic ceramic for three-unit prostheses involving molar restoration” or “a fully covered substructure for three-unit prostheses involving molar restoration” (class 4 materials). ISO 6872 further extends the unlimited (all-in-one) clinical use as a “monolithic ceramic for prostheses involving four or more units or fully covered substructure for prostheses involving four or more units” (class 5 materials) for materials offering a flexural strength of more than 800 MPa.

The aim of this study was to analyze the flexural strength of different layers of representative 4/5Y-PSZ zirconia multilayer materials under three-point bending conditions and to assess their strength potential in clinical applications.

## 2. Materials and Methods

### 2.1. Materials

The products used in this investigation are listed in Table 1 and specified in Table 2. All materials are strength-gradient multilayer discs for labside use, consisting of dentin, several transition and enamel layers with 4 mol% yttria-partially stabilized zirconia (4Y-PSZ) in the dentin, and 5 mol% yttria-partially stabilized zirconia (5Y-PSZ) in the enamel layer. All discs offered a consistent thickness of 18 mm, except the material PE (which was only available in 20 mm). The clinical indications vary, as IPS e.max ZirCAD Prime Esthetic is indicated for three-unit prostheses involving molar restorations (class 4 according to ISO 6872), while all other materials are marketed for unlimited use (class 5).

### 2.2. Specimen Preparation

The test specimens were prepared from the zirconia discs via computer-aided design/computer-aided manufacturing (CAD/CAM; Brain CAM Pro V3.3.021, Dentsply Sirona). Tetragonal bars with dimensions 25 × 4 × 3 mm were constructed using CAD software (Blender V4.3, Blender Foundation, Amsterdam, The Netherlands), and the *.stl dataset was used for CAM machining in a Brain Expert (Dentsply Sirona) milling device.

**Figure 1:**
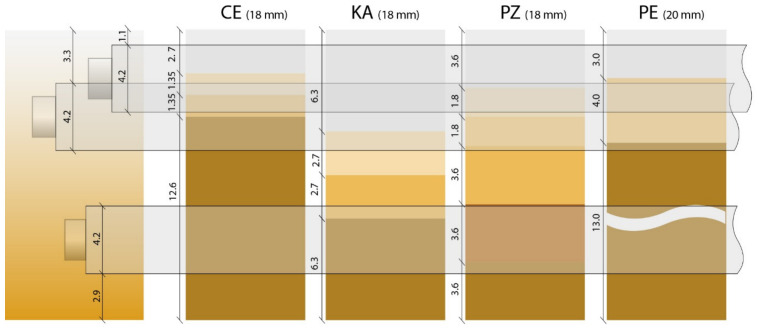
Nesting of specimens in the discs in relation to the individual layer concept (colors are used to distinguish between layers from light (inc) to drak (den)).

The specimens were nested in the discs (13 specimens per disc, 3 discs (n > 30 per group)). The experimental groups were nested to reflect the different layers in a zirconia disc (Figure 1). The upper plane of a specimen (compression side in later flexural testing) was placed either 1.1 mm away from the upper plane of the disc (nested in the incisal layer (inc) or 3.3 mm away from the upper plane (nested in the transition layer (tra)). Regarding the dentin specimens (den), the bottom plane of a specimen (tensile side in later flexural testing) was placed 2.9 mm away from the bottom plane of the discs. In total, 360 specimens were tested.

**Figure 2:**
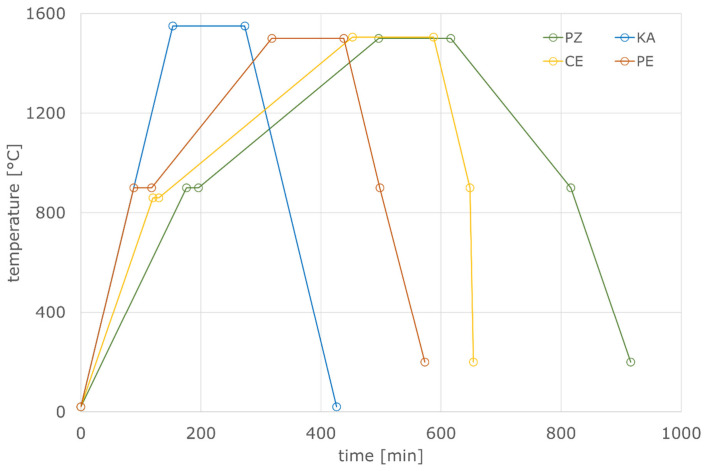
Firing cycle durations for the materials under investigation.

All specimens were sintered to final densities, using a Zyrcomat 6000 MS furnace (Vita Zahnfabrik, Bad Säckingen, Germany) in accordance with respective manufacturer recommendations. The firing schedules are outlined in Table 3, and the duration is plotted in Figure 2 (recommendations for long-span units (CE: >8 units; KA: prog 1; PZ: >7 units; PE: standard prog).

### 2.3. Specimen Finishing and Polishing Procedure

After sintering, the test specimens were further processed to ensure certain dimensions (4.0 ± 0.2 mm (width), 3.0 ± 0.2 mm (thickness), and 25 mm (length)), plan-parallelism, and a consistent surface quality. On all specimen faces, a Tegramin-25 semiautomatic polishing machine (Struers, Ballerup, Denmark) was used. The finishing protocol was based on the MD Piano system (resin-bonded diamond grinding discs; Struers). Sequences of rough (MD Piano 220) to fine (MD Piano 1200; equals P1200 SiC Paper) were applied under sustained water cooling, and specimens were finished until final dimensions were reached. Specimen flanges were bevelled by hand using P1200 SiC Paper (Struers). The tension faces of the specimens underwent an additional polishing step using the MD Plan/DiaPro All, together with a Largo 9 μm diamond suspension (all Struers). The tension face of the specimens was assigned to the bottom face of the specimens in the discs. The specimen dimensions were individually recorded using a digital calliper (Garant, Hoffmann SE, Munich, Germany; ±0.01 mm).

All specimens were stored in dark and dry conditions until fracture strength testing. The test environment had a temperature of 21 °C and a relative humidity of 35%.

### 2.4. Uniaxial Fracture Strength Testing in Three-Point Bending Configuration According to ISO 6872

**Figure 3:**
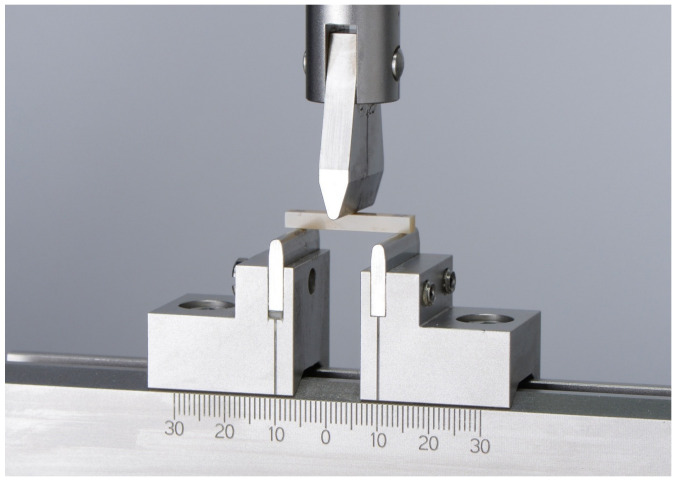
Semi-articulating fixture for uniaxial 3PB flexural strength testing with span length L = 20 mm.

For the flexural strength test in the three-point bending (3PB) configuration, a semi-articulating fixture was used, as shown in Figure 3. To support the test specimens, a span length of L = 20 mm was selected. The upper load-bearing was placed centrally to the support bearings.

Each group consisted of 30 specimens. The specimens were carefully positioned centrally and perpendicular to the load and support bearings’ axes in the test fixture, and the load was applied centrally to the support span.

A pneumatic universal testing machine (TP 5KN HF, DynaMess, Aachen, Germany) served as the testing device and was equipped with a 5.0 kN load cell (accuracy: class 1, ISO 7500-1). The specimens were fractured at a crosshead speed of 20 N/s.

The three-point bending fracture strength s was calculated according to [15] as follows:

(1) $$\sigma =\frac{3\cdot F\cdot L}{2\cdot b\cdot d^{2}}$$

where

σ is the fracture strength, in [MPa];

L is the load causing fracture, in [N];

F is the support span length, in [mm];

b is the specimen width, in [mm];

d is the specimen thickness, in [mm].

### 2.5. Statistical Analysis

The relation of fracture strength data to either a Gaussian (normal) or a Weibull distribution was tested using the Kolmogorov–Smirnov or the modified K-S D test, respectively. As the data under investigation fit both Gaussian and Weibull distributions, we treated our data to show the characteristic strength with regard to the failure probability and to present mean values to meet the requirements outlined in ISO 6872.

The widely used statistical model for brittle fracture is based on the weakest link hypotheses laid out by the Weibull theory, which computes a failure probability function P_F_ in a homogeneous stress state σ, in a specimen of volume V, according to

(2) $$P_{F}\left(\sigma ,V\right)=1-exp\left[-\frac{V}{V_{0}}{\left(\frac{\sigma }{\sigma_{0}}\right)}^{m}\right]$$

V_0_ and σ_0_ are the characteristic volume and characteristic strength values, respectively, which represent a probability of failure of P_F_ = 63.2% when V = V_0_ and σ = σ_0_. The exponent m is the Weibull modulus and describes the scatter of strength values (homogeneity parameter). The Weibull scale (σ_0_) and the shape (m) parameters are derived from a ln(σ_0_) versus ln(ln(1/(1 − P_F_))) plot. The procedure is described in ISO 20501 [20].

Mean values and standard deviations of the fracture strength data were further statistically analyzed using two-way ANOVA (factors: “layer”, “material”) and S-N-K post hoc test at a significance level of α = 0.05.

### 2.6. Microstructure and Fractographic Analysis

To distinguish among the incisal, transition, and dentin layers, confirm the sintering density, and to exclude residual porosity, exemplary microstructural images from the material CE were analyzed using a field-emission scanning electron microscope (FESEM, Auriga, Zeiss, Oberkochen, Germany). The tensile face (polished surface) of the fractured specimens was thermally etched for 30 min at 1150 °C. Additionally, selected fractured specimens were fractographically analyzed (high- versus low-fracture-strength samples) in order to locate the fracture origins, determine the respective reasons for fracture, and identify possible outliers (fractured specimens of low strength that fractured due to invalid fracture causes).

## 3. Results

### Same Superscript Letters Indicate Statistically Homogeneous Subgroups

**Figure 4:**
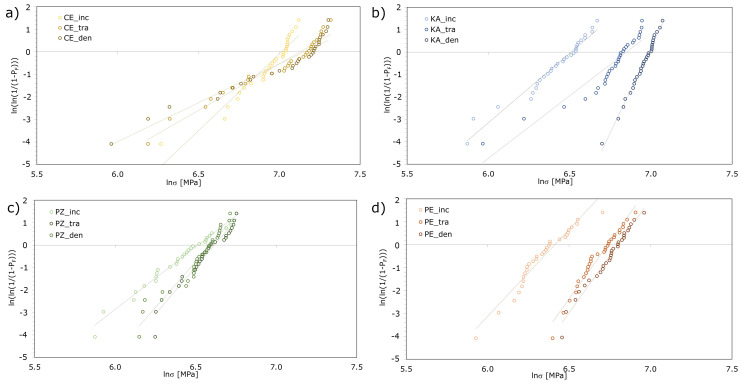
Weibull plots of the four materials ((**a**): CE; (**b**): KA; (**c**): PZ; (**d**): PE), comparing the three different layers.

Table 4 summarizes the results from the flexural strength testing. Mean values and standard deviations are presented, and statistically homogeneous subgroups are indicated using superscript letters. Furthermore, characteristic fracture strength σ_0_ data along with their Weibull moduli m and respective 90% confidence intervals are displayed. Figure 4 a–d display the Weibull distributions of the four investigated materials, comparing each of the three layers. Two-way ANOVA revealed a statistically significant influence of the factors “layer” (*p* < 0.01) and “material” (*p* < 0.01), as well as “layer x material” (*p* < 0.01). Student-Newman-Keuls post-hoc showed significant differences for the “inc,” “tra,” and “den” groups, as well as among the four test materials. Statistically homogeneous subsets are labelled(superscripted uppercase letters) in Table 4.

In general, the highest flexural strength level was reached in the dentin layers of all materials, with the CE_den group exhibiting the highest mean flexural strength of σ = 1142.73 MPa. All CE layers produced a statistically homogeneous subset, and CE_inc achieved a mean strength of σ = 1036.87 MPa. This level of strength was only reached by the KA_den group (σ = 1045.97 MPa). For all other materials, a significant strength reduction was observed in the incisal layers. The lowest mean flexural strength was measured for the PE_inc (σ = 571.97 MPa), being statistically homogeneous with the KA_inc (σ = 622.50 MPa) and PZ_inc (σ = 623.82 MPa) groups. The material PZ showed only a slight increase in strength from incisal to dentin layers, with no statistically significant differences between the PE_tra and PE_den layers. The materials KA and PE showed a statistically significant strength increase from incisal to dentin layers.

For the flexural strength distribution results, the scatter of data (standard deviation) and reliability (Weibull moduli) are reported in Table 4. Among all test groups, a maximum reliability of m = 16.5 was achieved in the KA_den group, while CE-den showed the lowest Weibull modulus of m = 4.7. In particular, the three lowest data points in group CE_den (#14: σ = 555.86 MPa; #21: σ = 387.98 MPa; #27: σ = 486.88 MPa), compared in Figure 4a, were responsible for the low reliability of the CE_den group and hence received special attention in the fractographic analysis. The reliability of the incisal layers was intermediate, offering Weibull moduli of m = 9.4/6.5/5.5/7.3 for the materials CE_inc, KA_inc, PZ_inc, and PE_inc, respectively.

**Figure 5:**
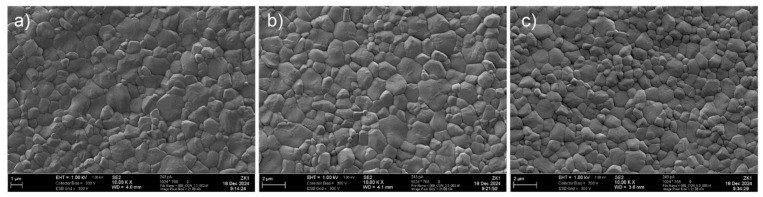
Microstructural SEM images of the material CE, showing the incisal (**a**), transition (**b**), and dentin (**c**) layers. A pore-free microstructure in all layers and only a slightly increased grain size from 3Y-PSZ (**c**) to 5Y-PSZ (**a**) can be observed in CE.

The fracture strength gradient for layers from incisal to dentin regions is closely related to the microstructure of the materials. Figure 5a–c show the grain size distribution of each layer, for the example of CE_inc, CE_tra, and CE_den under SEM. The larger grain size of the 5Y-PSZ material (up to 2 µm in diameter) is shown in Figure 5a. Figure 5b offers a mixture of smaller and larger grains, indicating the 4Y-PSZ along with the 5Y-PSZ fraction. Figure 5c shows the 4Y-PSZ grain size distribution of CE-den with grain sizes of <1 µm.

**Figure 6:**
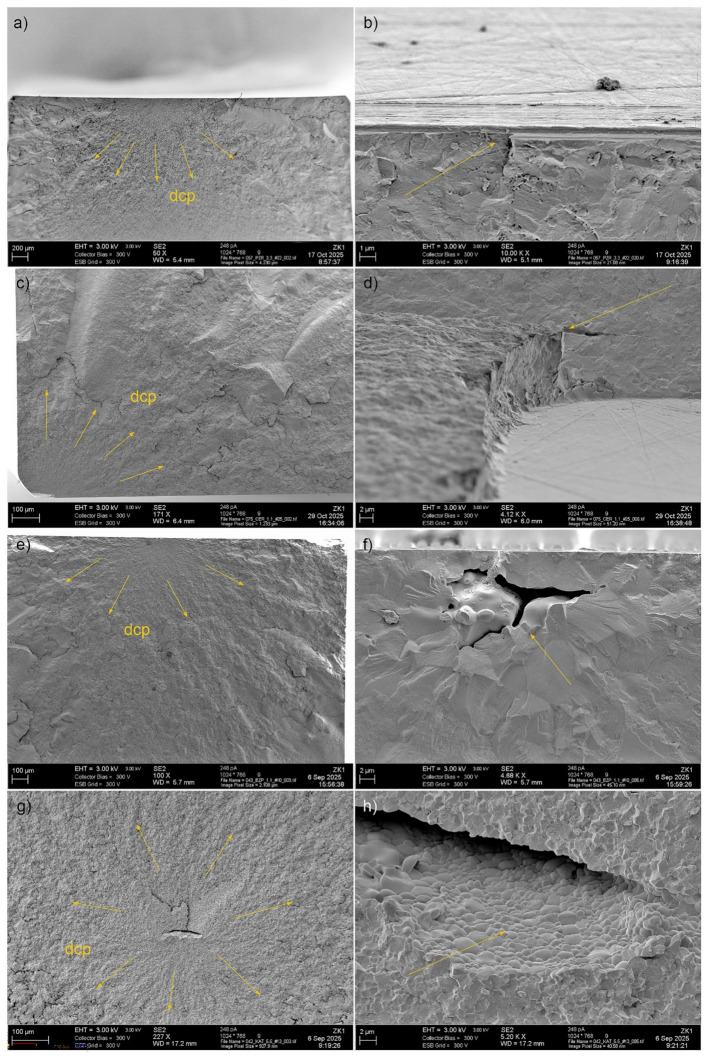
Typical fracture origins and related reasons for fractures analyzed using fractographic SEM-microscopy: (**a**,**b**): example of a surface grinding defect; (**c**,**d**): example of a specimen processing edge defect; (**e**,**f**): small, subsurface sintering defect; (**g**,**h**): large internal pressing defect (dcp: direction of crack propagation, arrows indicate either respective dcp or the fracture origin).

Fractured specimen inspection, especially with regard to fracture origin and reasons for fracture, is an important analytical tool for distinguishing material- versus processing-related fractures and assigning typical fracture patterns to resulting characteristic strength data. For that reason, the fracture surfaces of selected specimens from all groups were first screened under a stereo microscope (Discovery V.8, Zeiss) and typical fracture patterns were identified. A deeper fractographic analysis of selected specimens was performed using SEM, especially for specimens assigned to low-strength data from each group. Figure 6a–h show examples of typical fracture-releasing defects found in all materials. Figure 6a,b provide an indication of a surface-located grinding defect (PZ_tra group: #22, σ = 517.29 MPa). The fracture origin is typically found in a deep surface grinding groove. Figure 6c,d show a fracture origin located at the specimen flange (CE_inc group: #25, σ = 848.16 MPa). This fracture type is also a typical reason for fracture, arising from a grinding defect, close to the specimen edge. Most cases of this fracture type are prevented by beveling the specimen flanges before testing. In contrast, Figure 6e–h are related to pressing defects, already inherent to the material white-bodies before sintering. Figure 6e,f indicate a small pore of approx. 10 µm in diameter, located in the high-tensile stress, subsurface region of the specimen (PE-inc: #10, σ = 543.85 MPa). Figure 6g,h show an internal pressing defect of larger size (KA_den: #13, σ = 1051.48 MPa), which is located away from the maximum tensile stress region; however, due to the larger extent (approx. 100 µm in diameter), this defect was assigned as a fracture-releasing defect.

Apart from the typical fracture patterns, no abnormal fracture patterns were found in the low-strength specimens (e.g., CE_inc group) that would have led to the exclusion of data points from the analysis.

## 4. Discussion

This study investigated the flexural strength of four 4Y/5Y-PSZ zirconia multilayer disc materials, according to the procedure outlined in ISO 6872. Significantly different strength levels have been observed within the various layers in each material, as well as among competitor products.

As an internationally accepted minimum requirement for clinical use, ISO 6872 proposes a mean flexural strength of >800 MPa as the threshold for long-span (monolithic or veneered) bridgework (more than four units) intended for so-called “universal” or “all-in-one” use [15]. Based on this benchmark, only the material CE offers unlimited clinical use throughout all layers. The materials KA and PE partly fulfil this criterion, as the flexural strength of only the 5Y incisal layers falls below this threshold value. The material PZ does not reach the level of 800 MPa in any layer. A comparison of the measured data here with the manufacturer data reported in Table 2 shows a different classification. While the flexural strength of the materials CE, KA, and PE well correlates with manufacturer data, the mean values for PZ are very different. The measured data for the material CE exceeds the manufacturer data in all layers, while KA shows a consistent trend, with the manufacturer data with the 5Y incisal layer falling below the ISO threshold (622 versus 750 MPa, as reported by the manufacturer). The performance of the material PE in principle follows the manufacturer data in the incisal layer (572 versus 650 MPa), as well as in the dentin layer (853 versus 850 MPa). Only the material PZ is advertised by the manufacturer for all-in-one use, but the results under investigation did not confirm the mandatory flexural strength, neither in the incisal layer (624 versus >800 MPa) nor in the dentin layer (686 versus > 1100 MPa). The underlying reasons can only be speculated, as the experimental procedure of this material was in line with all other products, and the microstructure did not account for an increased defect population. Only sparse information on applied testing protocols is disclosed by this manufacturer.

Within the dental community and through years of mechanical research, the three-point bending configuration has manifested its position as the key predictor of ceramic strength. Today, more types of uniaxial and multiaxial strength testing routines have been established and even found their way into the ISO 6871 standard. An apparent limitation to the ISO 6872 threshold strength values, however, is the unspecific assignment of the testing routine. ISO 6872 recommends flexural strength testing in either a uniaxial setting (three-point-bending and four-point-bending) or a biaxial configuration (piston-on-three-balls). It is known that different testing procedures produce different strength levels, as the maximum loaded volume in a specimen varies [21]. As a practical consequence, the absolute data measured in biaxial testing produces significantly higher values compared to three-point bending or four-point bending (biax > 3-PB > 4-PB). The data in Table 4, as well as the manufacturer data for CE, KA, and PE, were measured using a three-point bending setup. One manufacturer (KA), however, used specimens of dimension 3 × 4 × 40 mm, different from the employed dimensions of 3 × 4 × 25 mm. The method used for PZ is not disclosed by the manufacturer. In case the manufacturer data is derived from a biaxial approach, the divergence from our PZ data might be assigned to the testing routine.

The scientific literature provides reference data for the materials under investigation; in particular, KA and PE are well-documented materials. For KA, Inokoshi et al. measured a value of 634 MPa in the incisal (measured in a four-point bending configuration) and 911 MPa in the dentin layer [22]. They also measured a biaxial flexural strength of 748 MPa (incisal) and 1074 MPa (dentin). Another contribution measured a biaxial mean flexural strength of 849/922/1010 MPa in the inc/tra/den layers [23]. As the material CE is new to the dental market, no strength-related data were found in the literature. However, it is indicated that new powder technology enables high-strength performance along with consistently high esthetics, intended for use in the incisal part of multilayer discs. A new primary zirconia powder source has recently been introduced and characterized [24]. Nakai et al. measured a characteristic biaxial flexural strength of 978 MPa for such a material, which shows good consistency with the measured data under investigation (1037 MPa, measured in a three-point bending configuration). For the product PZ, no respective literature data were accessible.

Along with strength testing, the data scatter is a matter of interest. The scatter of values measured in a population of 30 specimens is considered representative for the assessment of homogeneity. Weibull analysis offers insight into characteristic strength as a function of failure probability. Such factorial dependence is expressed by the Weibull modulus m. Table 4 offers a broad range from m = 4.7 (CE_den) up to m = 16.5 (KA_den). The slope of the regression line in Figure 4a–d confirm the variations in material homogeneity. A low homogeneity is often associated with large defects in low-strength specimens. Such possible outliers (e.g., see the bottom data points in Figure 4a–d) in a Weibull plot are commonly inspected using fractographic post-processing. Figure 6a–h show examples of typical defect types taken from the materials under investigation, such as finishing defects (surface/edge grinding) or processing defects (small pores or pressing defects). The detection of abnormally large or unusually shaped defect types might lead to exclusion from the Weibull analysis, in turn, elevating the Weibull modulus. For the materials under investigation, however, no unusual defect types were confirmed, and all specimens were kept for further statistical analysis. In fact, the specimen preparation strategy has a strong influence on the experimental outcome, both regarding the mean strength and the scatter of the resulting data. Lab specimen processing is generally different from clinical procedures, and hence, the data have only limited clinical transferability [25]. The approach in this study used a practical, relevant CAD/CAM machining strategy to produce the precursor specimens, but the surfaces after sintering were further finished and polished using lab-specific techniques.

Overall, the flexural strength measured for the materials under investigation guides their clinical indication. The products KA and PE are limited in their clinical application. For KA, the manufacturer excludes class 5 use with a connector located in the incisal layer [17]. The material PE is entirely restricted by the manufacturer to class 4 use, prohibiting its clinical use in full-arch or long-span, all-in-one bridgework, although the material fulfils the ISO 6872 requirements in the PE_tra and PE_den layers [19]. For the all-in-one indication, the manufacturer offers the material IPS e.max ZirCAD Prime, a 3Y/5Y-PSZ zirconia multilayer material with a higher level of flexural strength. The material PZ, in contrast, is marketed for unlimited use [18]. However, the data reported here do not support this recommendation. The only material for unlimited all-in-one clinical use is CE, at least based on the measured flexural strength data.

For a clinical consideration of the precursor discs, the respective layering concept is of interest, as the position of the designed bridge connectors may vary between products according to layer thickness. Figure 1 displays the layer concepts as disclosed by the manufacturers. As per beam theory, the bottom face of a specimen is under maximum tensile stress, and fracture is expected to emanate from this location. Upon clinical occlusal forces, this translates into a gingival bridge connector face under maximum tensile stress. The nesting of the dentin specimens in the discs is not of concern, as all products consist of a thick dentin layer (with the maximum tensile stress region in the dentin zone). However, the nesting of specimens in the incisal layers leads to major differences, as incisal layer thickness varies significantly among products. Figure 1 shows the position of the test specimens in the respective precursor discs. While all dentin specimens are entirely nested in the dentin layers, the situation for the incisal specimens is different. For KA only, the tensile face of the specimen is located in the incisal layer (6.3 mm thickness); for all other products, this face is positioned partly in the transition layers. Regarding the transition specimens, CE is located in the dentin layer, while PZ and PE are slightly touching the dentin layer. The tensile face of KA_tra specimens is located in the transition zone. The situation for PZ is not clear, as this material is reported to consist of six different layers. This observation partly explains why the flexural strength data for CE, PZ, and PE between the transition and dentin layers are insignificant. Only KA shows significant differences between KA_tra and KA_den.

In conclusion, one could raise the question of why the all-in-one material class consists of 4Y/5Y-PSZ zirconia multilayer gradients instead of 3Y/5Y-PSZ multilayers, as the reported mechanical performance of 3 mol% yttria-stabilized zirconia still exceeds the level of 4Y or 5Y-PSZ materials. Conventional 3Y-TZP zirconia typically exhibits flexural strength values up to 1200 MPa and fracture toughness values ranging from approximately 4–5 MPam^0.5^. In contrast, high-translucency zirconia materials such as 5Y-PSZ generally show flexural strengths between 600 and 800 MPa and a lower fracture toughness of approximately 2–3 MPam^0.5^ due to the reduced contribution of transformation toughening [8,26,27]. The answer is ascribed to two factors: first, the esthetic clinical outcome of 4Y-PSZ is superior compared to 3Y-TZP, even in the relatively opaque dentin layer; and second, constrained sintering of gradient multilayer materials is a challenging production step.

Increasing the yttria concentration from 3 mol% to 4 or 5 mol% results in the stabilization of a greater fraction of the cubic phase. Cubic grains are optically isotropic and exhibit reduced birefringence compared with tetragonal grains, which significantly reduces light scattering and improves translucency [7,28]. The relative proportion of tetragonal and cubic phases strongly influences both mechanical and optical properties. Tetragonal grains enable transformation toughening, whereas cubic grains improve translucency but do not contribute to this toughening mechanism. Consequently, increasing the cubic phase fraction improves esthetics but reduces fracture toughness and flexural strength [8]. As a result, high-translucency zirconia materials have been introduced that provide significantly improved optical performance while maintaining adequate mechanical properties, even for class 5 use as all-in-one materials.

Furthermore, high-translucency zirconia systems based on 4Y-PSZ and 5Y-PSZ have enabled the use of zirconia in indications previously dominated by glass–ceramic materials. These include monolithic anterior crowns, short-span bridges, and restorations even in the esthetically demanding anterior region.

Although multilayer zirconia provides improved esthetic outcomes, the presence of compositional gradients can introduce complex microstructural and mechanical interactions during sintering and subsequent cooling. Differences in densification kinetics (i.e., sintering shrinkage and shrinkage rate) between layers with varying yttria contents may lead to differential shrinkage during sintering [29]. Constrained sintering of layers is a balanced function of particle grain size (cubic grains > tetragonal grains) and residual porosity distributions, hence defining state densities and, in turn, sintering kinetics [30]. When shrinkage is constrained by adjacent layers or by the geometry of the restoration, internal stresses can develop. These residual stresses, particularly effective at layer interfaces, may influence the mechanical properties and dimensional trueness of the final FPDs [31]. The practical answer to such constraints is to produce a gradient instead of discrete transitions between layers.

Physical testing of restorative materials is not strictly related to ISO protocols but should be further amended toward clinical relevance. Future studies should involve cyclic (intraoral) loading or thermocycling protocols, which could both have a significant influence on clinical longevity.

## Figures and Tables

**Table 1:** Details of 4/5Y-PSZ zirconia disc materials under investigation.

	Cercon yo ML	Katana Zirconia YML	3D Pro Zir	IPS e.max ZirCAD Prime Esthetic
Manufacturer	Dentsply Sirona (Hanau, Germany)	Kuraray Noritake (Aichi, Japan)	Aidite (Qinhuangdao City, China)	Ivoclar (Schaan, Liechtenstein)
Label	CE	KA	PZ	PE
Shade/height	A4/18 mm	A4/18 mm	A4/18 mm	A4/20 mm
LOT	18057921	EOGLO/EPRNR	56957900/59543700	YB737K/YB72VY
REF	5366105418	125-8063	APW9818-3DMA4	752142

**Table 2:** Multilayer composition and properties of the materials under investigation (manufacturer data).

	Cercon yo ML [16]	Katana Zirconia YML [17]	3D Pro Zir [18]	IPS e.max ZirCAD Prime Esthetic [19]
Layer concept	Incisal: 2.7 mm (15%) Transition 2: 1.35 mm (7.5%) Transition 1: 1.35 mm (7.5%) Dentin: 12.6 mm (70%)	Incisal: 6.3 mm (35%) Transition 2: 2.7 mm (15%) Transition 1: 2.7 mm (15%) Dentin: 6.3 mm (35%)	Layer 1: 3.6 mm (20%) Layer 2: 1.8 mm (10%) Layer 3: 1.8 mm (10%) Layer 4: 3.6 mm (20%) Layer 5: 3.6 mm (20%) Layer 6: 3.6 mm (20%)	Incisal: 3.0 mm (15%) Transition: 4.0 mm (20%) Dentin: 13.0 mm (65%)
Flexural strength [MPa], ISO6872	Incisal: 900 Dentin: 1100	Incisal: 750 Dentin: 1100	Incisal: ≥800 Dentin: ≥1100	Incisal: 650 Dentin: 850
Translucency	Incisal: 49% Dentin: 45%	Incisal: 49% Dentin: 45%	Incisal: 57% Dentin: 43%	Incisal: n.a. Dentin: n.a.
Indication range	Class 5	Class 5	Class 5	Class 4
Design recommendations (posterior)	min. wall thickness: 0.5 mm min connector: 9 mm^2^	min. wall thickness: 1.0 mm min connector: 9 mm^2^	min. wall thickness: 1.0 mm min. connector: 12 mm^2^	min. wall thickness: 1.0 mm min connector: 16 mm^2^

**Table 3:** Firing regimes for the materials under investigation.

	Temperature [°C]	Heating Rate [°C/min]	Holding Time [min]	Cooling Rate [°C/min]
Cercon yo ML [16]	20–860	7		
	860		10	
	860–1505	2		
	1505		135	
	1505–900			10
	900–200			120
Katana Zirconia YML [17]	20–1550	10		
	1550		120	
	1550–20			10
3D Pro Zir [18]	20–900	5		
	900		20	
	900–1500	2		
	1500		120	
	1500–900			3
	900–200			7
IPS e.max ZirCAD Prime [19]	20–900	10		
	900		30	
	900–1500	3		
	1500		120	
	1500–900			10
	900–300			8

**Table 4:** Fracture strength data FS (± SD), m, and σ_0_, including respective 90% confidence intervals.

	Mean Flexural Strength (±SD) [MPa]	Weibull Modul m	90% Confidence Interval	Characteristic Strength σ_0_ [MPa]	90% Confidence Interval
CE_inc	1036.87 ± 157.7 ^A^	9.4	7.0–11.4	1097.04	1058.57–1137.35
CE_tra	1131.43 ± 284.7 ^A^	5.2	3.9–6.3	1236.02	1158.53–1319.63
CE_den	1142.73 ± 316.9 ^A^	4.7	3.5–5.8	1254.10	1168.37–1347.19
KA_inc	622.50 ± 109.9 ^C,D^	7.3	5.5–8.9	665.60	635.85–697.09
KA_tra	867.84 ± 147.7 ^B^	8.5	6.3–10.3	921.50	885.82–959.02
KA_den	1045.97 ± 82.1 ^A^	16.5	12.3–20.1	1080.85	1059.15–1103.24
PZ_inc	623.82 ± 131.6 ^C,D^	5.5	4.1–6.7	676.05	636.16–718.92
PZ_tra	704.86 ± 99.6 ^C^	8.4	6.3–10.3	747.06	718.07–777.57
PZ_den	685.71 ± 93.2 ^C^	9.8	7.3–11.9	723.07	698.77–748.50
PE_inc	571.97 ± 91.7 ^D^	6.5	4.8–7.9	611.00	580.22–643.78
PE_tra	802.02 ± 97.7 ^B^	9.3	6.9–11.3	844.71	814.80–876.07
PE_den	853.62 ± 102.5 ^B^	10.0	7.4–12.2	897.17	867.00–928.75

## Data Availability

The original contributions presented in the study are included in the article, further inquiries can be directed to the corresponding author.
